# Pigeon egg white protein-based transparent durable hydrogel via monodisperse ionic surfactant-mediated protein condensation

**DOI:** 10.1038/s41598-022-08375-x

**Published:** 2022-03-17

**Authors:** Xinlian Zhou, Zaozao Chen, Tatsuya Nojima

**Affiliations:** State Key Laboratory of Bioelectronics, School of Biological Science and Medical Engineering, Southeast University, Nanjing, 210096 China

**Keywords:** Biomaterials - proteins, Biomaterials - proteins, Gels and hydrogels

## Abstract

The thermal gelation property of proteins is useful in creating protein-based materials. The gelation of protein solution often proceeds by the random aggregation of denatured proteins, and the protein-based gels are typically brittle or opaque, or both. Improvement in the mechanical and optical properties of protein-based materials are required for them to be practical and functional. This study investigated pigeon egg white, which is semitransparent in its thermally gelled state, as a protein source for creating hydrogel materials. The protein thermal gelation process was initiated from the orderly condensed state of proteins complexed with monodisperse ionic surfactants to suppress random aggregation. The resultant gel showed transparency in the visible light region and was not destroyed at 99% compression under 17.8 MPa compressive stress, 350-fold higher than the compressive fracture strength of typical boiled pigeon egg white. These results showed that durable transparent hydrogels could be fabricated by the rational combination of natural proteins and surfactants.

## Introduction

Protein materials have attracted attention in modern material science owing to their functional tunability and sustainable productivity. Natural proteins, such as silk, collagen, and gelation, and protein-polymer conjugates have been used to create protein-based functional, practical protein hydrogels with advantages such as biocompatibility and degradability for use in biomedical and cell biology applications^1–5^.

Mechanical strength and durability of the gel are necessary to produce micro- to macro-scale structures for practical applications. A simple way to increase the mechanical strength of the gel is to increase the concentration of its constituents; however, there is a limit to the increase. Additionally, increasing the constituent concentrations often leads to turbidity, decreasing its optical transparency, which in some applications such as tissue-engineered construct, is necessary to observe the internal aspects of the fabricated gel structure. The mechanical strength of the gel could also be increased by improving the internal structural order of the gel network. The mechanical properties of hydrogels with well-designed, ordered network structures are superior to those of typical hydrogels fabricated by the random polymerization process^6,7^.

Eggs are one of the most ubiquitous foodstuffs worldwide. Egg white is rich in protein, and its thermal gelation is a well-known property observed when cooking eggs. Because thermal denaturation and aggregation of the denatured proteins occur randomly during the heating process^8–10^, boiled egg white is a hydrogel with a heterogeneous network structure and low mechanical properties.

Previously, we reported a novel method for creating protein-based hydrogels with improved mechanical strength from hen egg white (HEW)^11^. In this process, HEW proteins were thermally gelled from the orderly condensed state of protein molecules termed “Protein Condensate (PC)^12^.” PCs are formed as fluid material phase-separated from aqueous by mixing both anionic and cationic surfactants that possess alkyl and polyethylene glycol (PEG) chains with the protein solution in a specific ratio. The electrostatic associations of proteins and surfactants results in the formation of a protein-surfactant complex, in which hydrophilic PEG and hydrophobic alkyl chains are located at the inner and outer side against the protein, respectively. The complexes assemble through alkyl-alkyl interactions to form PC as a transparent fluid, phase-separated from the water phase. The surfactants surrounding the proteins serve as spacer layers, and the proteins are spontaneously arranged at regular intervals in an ordered manner. Owing to the water-holding properties of PEG chains, the constituent proteins of PC are in an aqueous environment and maintain their native structure and functions (Fig. 3a).

The constituent PC proteins are arranged in an orderly manner at regular intervals and in spatially confined situations. The random aggregation of thermally denatured proteins can be suppressed in the PC state, resulting in the formation of a protein hydrogel with improved mechanical properties. The gel formed, PC(HEW)-gel, appeared opaque white, similar to boiled hen egg white, but showed a 170-fold higher compressive mechanical strength than usual boiled egg white.

PC(HEW)-gel was sufficiently strong to construct structured materials; however, the opacity of the gel limited its application. Thus, the development of a transparent PC(HEW)-gel is of great interest to tissue engineers and biomaterials scientists. Therefore, we examined other natural protein sources showing a transparent appearance in the thermal gelation state to create a transparent PC-gel.

**Figure 1:**
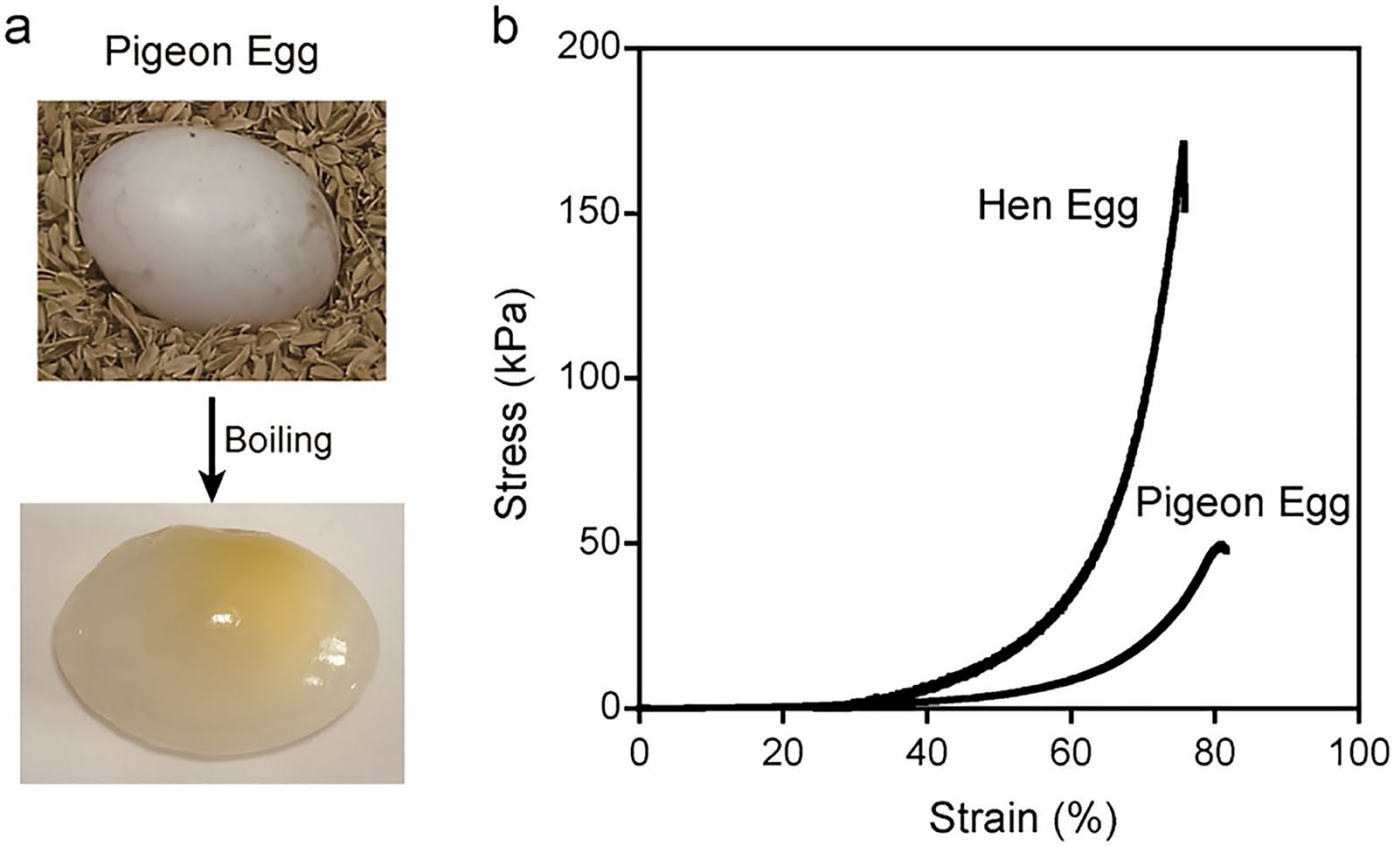
(**a**) Semitransparent appearance of boiled pigeon egg white (PEW); yolk is seen through it. Photos were taken by Xinlian Zhou. (**b**) Compressive stress–strain curves of boiled hen egg white and boiled pigeon egg white, showing the fracture strength of 170 kPa and 50 kPa, respectively.

Pigeons are widespread, resident-breeding birds raised for foodstuffs, and their eggs are available. Pigeon egg white (PEW) exhibits thermal gelation property similar to HEW. Interestingly, however, boiled PEW is semitransparent (Fig. 1a). This optical characteristic of boiled PEW inspired us to utilize PEW to create transparent PC-gel. Furthermore, we optimize the molecular structures of the ionic surfactants using for PC preparation to improve the mechanical characteristics of the gel. Here, we report a transparent hydrogel with high mechanical durability, created using PEW as a protein source combine with monodisperse ionic surfactants.

## Results

### Monodisperse surfactants in PC-gel preparation

**Figure 2:**
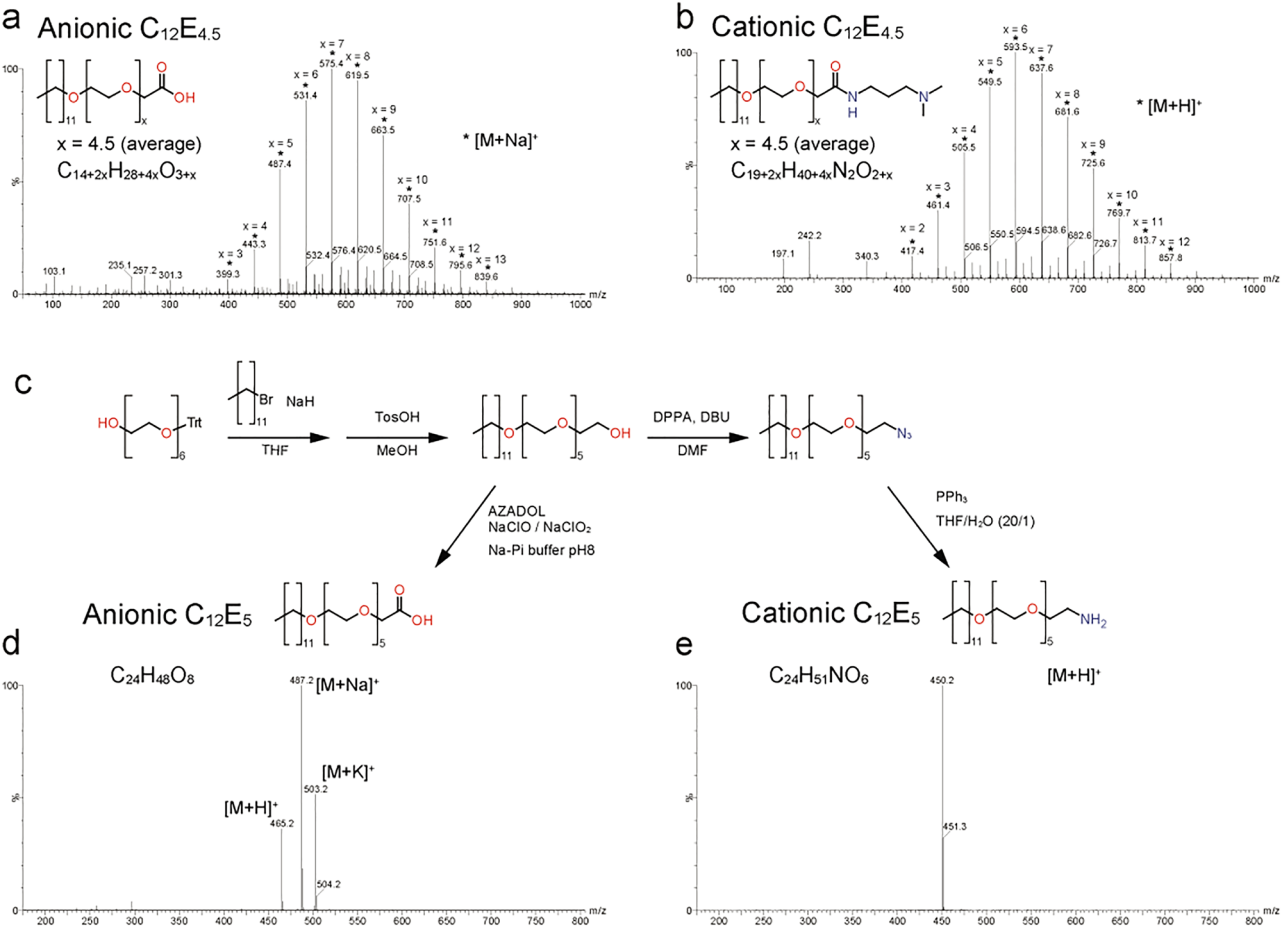
Monodisperse surfactants for the preparation of protein condensate (**a**,**b**) ES-MS spectra of polydisperse anionic and cationic C_12_E_4.5_ surfactants. C_12_E_4.5_ surfactants used in previous studies were mixtures with a repeating number of PEG units of 2–13. (**c**) synthesis route of monodisperse anionic and cationic surfactants (the details are described in supplementary information) (**d**,**e**) ES-MS spectra of monodisperse anionic and cationic C_12_E_5_ surfactants.

Boiled pigeon egg white has a softer texture and weak mechanical property on compression than boiled hen egg (Fig. 1b). Hence, we expected that the PC-gel prepared from PEW under the same preparation conditions used in previous study would show weaker mechanical properties than HEW PC-gel. The ordering character and arrangement of the constitutive protein of PC can be controlled by the molecular structure of the surfactants and affects the mechanical property of thermally formed PC-gels^11^. In a previous study, we used ionic surfactants prepared from commercially available surfactants with polydisperse PEG chain (Fig. 2a,b). PC is a complex material composed of proteins and surfactants. Since protein is a monodisperse-structured macromolecule, the polydispersity of the surfactants could be a dominant disruption factor in the homogeneity of the protein arrangement inside the PC. Therefore, we used monodisperse surfactants for PC preparation to reduce the heterogeneity of the components in PC for the fabrication of PEW protein-based PC-gel with improved mechanical properties. We synthesized monodisperse anionic and cationic C_12_E_5_ surfactants with dodecane chain and pentaethylene glycol chains based on a reported method for monodisperse polyethylene glycols synthesis (Fig. 2c–e)^13,14^. In this study, we used monodisperse ionic C_12_E_5_ surfactants and polydisperse ionic C_12_E_4.5_ surfactants as used in our previous study (4.5 is the average repeating number of PEG units determined by NMR)^12^ to prepare PC and PC-gels and compared their properties to elucidate the effect of the structural homogeneity of the surfactants on the mechanical characteristics of the PC-gels.

### Preparation and analysis of PCs from PEW solution

**Figure 3:**
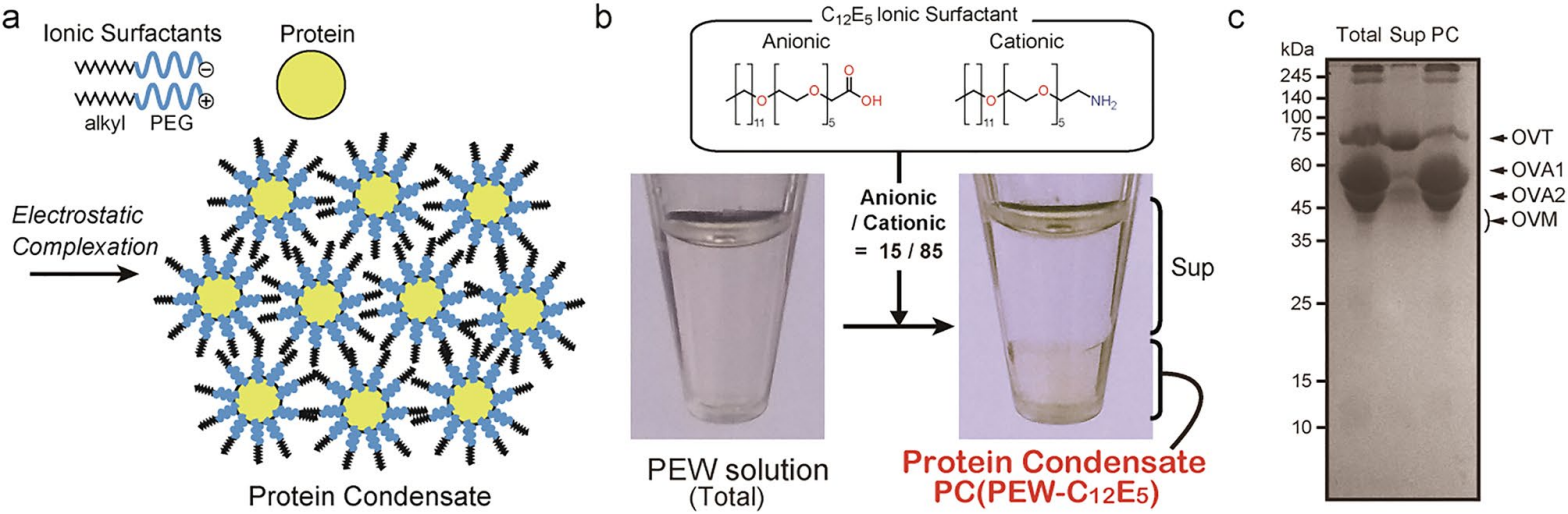
Protein condensate formation from pigeon egg white proteins (**a**) formation and structural model of protein condensate (PC) (**b**) PC from PEW proteins complexed with monodisperse cationic and anionic C_12_E_5_ surfactants, termed PC(PEW-C_12_E_5_). PC(PEW-C_12_E_5_) is formed as a transparent fluid, phase-separated from the upper aqueous layer (Sup). (**c**) SDS-PAGE analysis of proteins in PC(PEW-C_12_E_5_) (PC) and aqueous supernatant (Sup). “Total” indicates the PEW solution before adding surfactants. Major proteins are indicated (OVT, ovotransferrin; OVA1, ovalbumin 1; OVA2, ovalbumin 2; and OVM, ovomucoid)^15,16^.

We found that PC was formed from the PEW solution by adding a mixture of anionic and cationic C_12_E_5_ in an 85/15 ratio (Fig. 3b). In this study, we refer to PC formed using C_12_E_5_ surfactants as "PC(PEW-C_12_E_5_)." The PC(PEW-C_12_E_5_) protein and water contents were 138 mg mL^−1^ and 71% (w/w), respectively. The constitutive PC(PEW-C_12_E_5_) proteins were analyzed using SDS-PAGE (Fig. 3c). Ovalabumin (OVA) is the most abundant protein in egg white and exhibits thermal gelation property. HEW contains only one type of OVA, while PEW has two: OVA1 (53.2 kDa, also known as ovalbumin-related protein Y) and OVA2 (48 kDa)^15–17^. SDS-PAGE analysis showed that PC(PEW-C_12_E_5_) was mainly composed of OVA1 and OVA2. This result supported our idea that PC preparation using PEW as a protein source exhibits thermal gelation properties. The PEW PC with the C_12_E_4.5_ surfactant PC(PEW-C_12_E_4.5_) was also formed by adding a mixture of anionic and cationic C_12_E_4.5_ in an 85/15 ratio and showed a protein-based formation yield of 61%. The protein and water contents of PC(PEW-C_12_E_4.5_) were 137 mg mL^−1^ and 78% (w/w), respectively.

### Thermal gelation of PC(PEW-C_12_E_5_) and gel transparency

**Figure 4:**
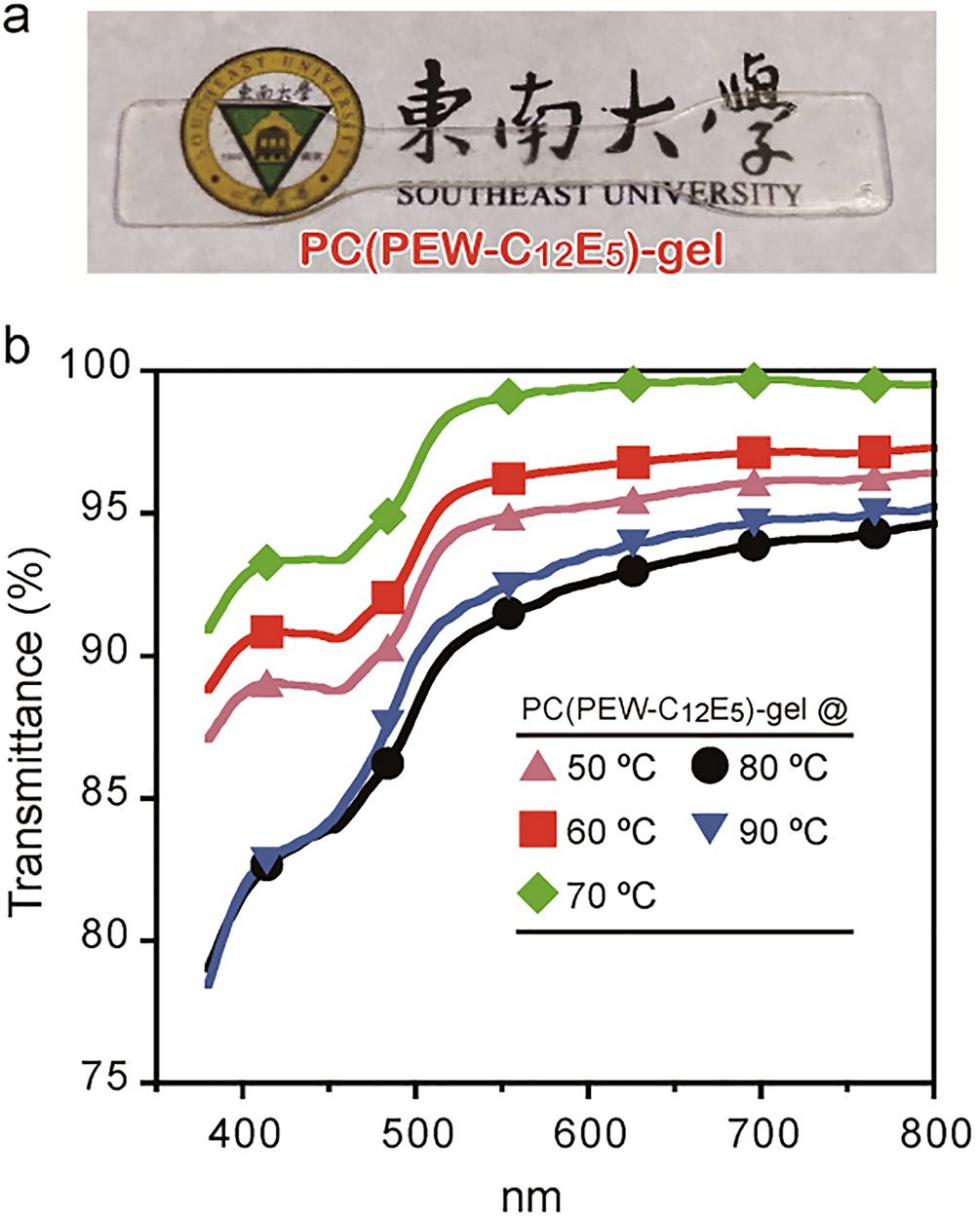
Pigeon egg white proteins based transparent gel (**a**) PC(PEW-C_12_E_5_)-gel was prepared by heating PC(PEW-C_12_E_5_) precursor. (**b**) Transparency of PC(PEW-C_12_E_5_)-gel (1-mm thick) prepared at various temperatures.

PC(PEW-C_12_E_5_) was gelled by heating it above 50 °C. We prepared PC(PEW-C_12_E_5_)-gels at 50, 60, 70, 80, and 90 °C. All PC(PEW-C_12_E_5_) gels were transparent in the visible light region (Fig. 4a,b). PC(PEW-C_12_E_5_)-gel prepared at 70 °C showed the highest transparency and was used in the following experiments. PC(PEW-C_12_E_4.5_) was also thermally gelled and transparent (Fig. S1). The results that both PC(PEW-C_12_E_4.5_)-gel and PC(PEW-C_12_E_5_)-gel showed the transparent appearance indicates that the optical transparency of the PC-gels is mainly derived from the natural properties of proteins used for PC preparation, rather than from the structure of the surfactant and protein arrangement inside PC that depends on the surfactant structure.

### Mechanical properties of the gels

**Figure 5:**
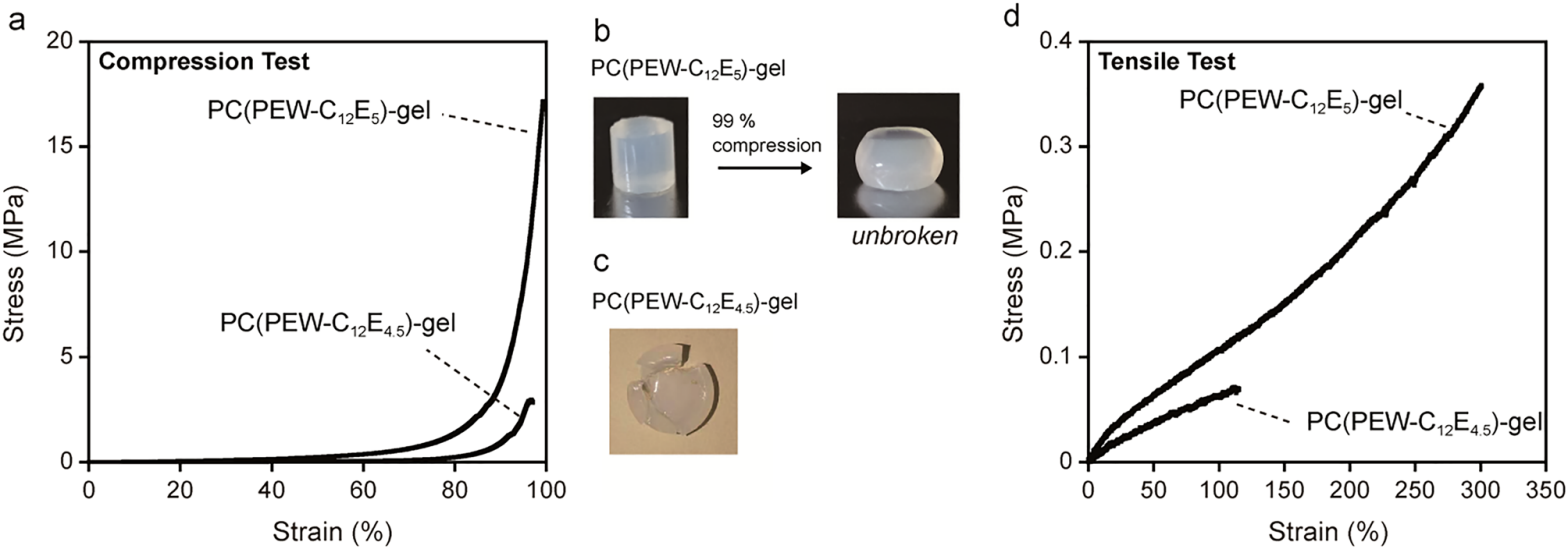
Mechanical properties of PC(PEW)-gel prepared with monodisperse C_12_E_5_ and polydisperse C_12_E_4.5_ surfactants prepared at 70 °C. (**a**) Compressive stress–strain curve. (**b**) PC(PEW-C_12_E_5_)-gel before and after 99% compression, showing non-destructive property against compression (**c**) destructed PC(PEW-C_12_E_4.5_)-gel after 96% compression (**d**) Tensile stress–strain curve.

The mechanical strength of PC(PEW-C_12_E_5_) and PC(PEW-C_12_E_4.5_)-gels was measured (Fig. 5). Both PC-gels showed improved mechanical properties compared to the boiled PEW broken at 50 kPa compressive stress (Fig. 1b). Notably, the mechanical properties of PC(PEW-C_12_E_5_)-gel were significantly superior to PC(PEW-C_12_E_4.5_)-gel. PC(PEW-C_12_E_5_)-gel was unbroken under 99% compression at 17.8 MPa compressive stress, while PC(PEW-C_12_E_4.5_)-gel was broken at 2.9 MPa (Fig. 5a–c). The nondestructive and durable property of PC(PEW-C_12_E_5_)-gel in a highly compressed condition were not observed in our previous research on PC gel of HEW prepared using polydisperse surfactants^11^. As shown in the tensile test, PC(PEW-C_12_E_5_)-gel also showed higher deformability and strength than PC(PEW-C_12_E_4.5_)-gel (Fig. 5d). These results supported our idea that the structural homogeneity of the PC state before thermal gelation is the determinate factor in the mechanical properties of the resultant PC-gel and can be improved using monodisperse surfactants in PC preparation. Following previous results^11^, these results demonstrated the versatility of the protein thermal gelation method from the PC state to improve the mechanical strength of protein-based hydrogel materials. The mechanical strength and non-destructibility of the gel under compression were not lost after washing to remove the surfactant, indicating that the improved mechanical properties of PC(PEW-C_12_E_5_)-gel originated from the network structure of the protein (see following section and Fig. S3).

### Biocompatibility of PC-gel

**Figure 6:**
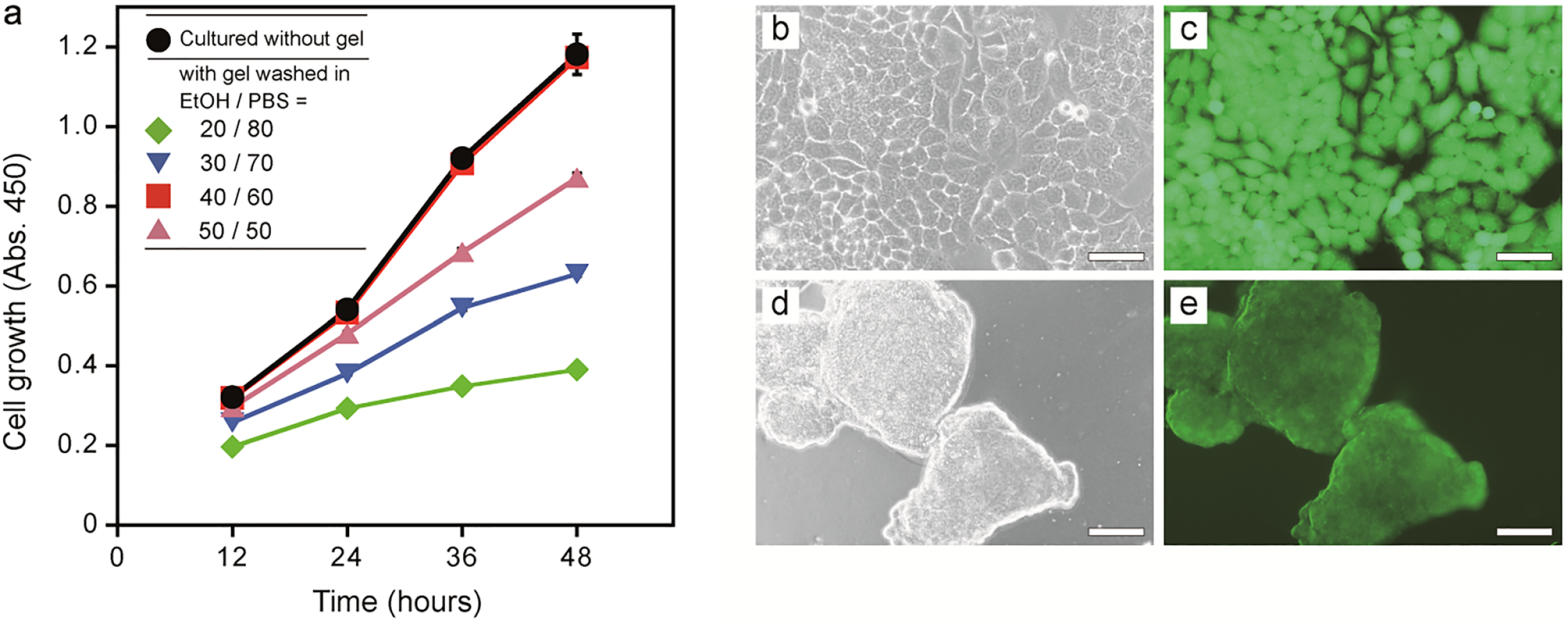
Biocompatibility of PC(PEW-C_12_E_5_)-gel and cell cultivation on the gel. (**a**) Growth of HL-60 cells in the presence of PC(PEW-C_12_E_5_) gels washed in ethanol and PBS the mixture. Washing with a 40/60 ethanol/PBS ratio (red filled square) showed normal cell growth equal to positive control group (cultured without gel, black filled circle). Error bars indicate S.D. (**b**–**e**) Growth of standard dish-cultured NCI-H460 cells (**b**,**c**) and on PC(PEW-C_12_E_5_)-gel washed in 40/60 ethanol/PBS (**d**,**e**). Bright-field microscope images of whole cells (**b**,**d**) and fluorescent microscope images of living cells (**c**,**e**). Scale bars: 200 μm.

Cell culture materials are a promising application for hydrogels owing to their water permeability, which is an essential property for the continuous supply of culture media^18^. PC(PEW-C_12_E_5_)-gel showed sufficient transparency for monitoring the culturing process and mechanical strength for future applications in fabricating structural cell culture scaffolds. We tested our PC gel as a cell-culture scaffold material. First, we performed a cytotoxicity assay to investigate the biocompatibility of the gel. HL-60 cells showed no growth under standard culture conditions in the presence of a piece of as-prepared PC(PEW-C_12_E_5_)-gel; however, the same assay performed with a piece of boiled PEW showed normal cell growth, suggesting that the ionic surfactants contained in the gel were cytotoxic and inhibited cell growth. Washing the surfactants from the prepared gel was required to safely apply PC gel in cell cultivation systems. Since alcohols and electrolytes inhibit hydrophobic and ionic interactions, respectively, we predicted that the combination of ethanol and PBS could be effective in removing ionic surfactants from PC gels (Fig. 6a). We found that cells can grow in the presence of the gels treated with the mixture of ethanol and PBS; a 40/60 ethanol/PBS ratio was most effective to render PC(PEW-C_12_E_5_)-gel non-cytotoxic. This PC(PEW-C_12_E_5_)-gel washing protocol was also effective for NCI-H460 cell growth (Fig. S2). In compression tests, washed PC(PEW-C_12_E_5_)-gel exhibited mechanical and nondestructive properties similar to the as-prepared gel (Fig. S3).

### Cell growth on PC-gel

Finally, we investigated the morphology of cells grown on PC gel. NCI-H460 cells were cultured on planar PC(PEW-C_12_E_5_)-gel and in a standard dish as a control. The control cells adhered to and propagated on the dish surface (Fig. 6b,c); whereas, the cells on planar PC(PEW-C_12_E_5_)-gel as a substrate supporting material formed living cell aggregates that did not adhere to the gel (Fig. 6d,e). The non-adhesiveness, improved strength, and transparency of PC gel could be advantageous in biocompatible structured scaffold material fabrication for three-dimensional cell culture used in preparing spherical, multicellular aggregates (spheroids) which could be utilized for biological application such as tissue engineering, developing of Organs-on-a-Chip, and as a model system for cell self-organization and metabolism research^19^.

## Discussion

This study was inspired by the transparency of thermally gelled PEW (boiled egg state). Although the molecular-level origin of the transparency has not yet been investigated, PEW proteins have been used as a protein source in creating hydrogels with improved optical transparency. It was shown that thermally treated PEW protein at PC state formed with newly synthesized monodisperse surfactants resulted in a transparent durable hydrogel with highly improved mechanical properties that showed nondestructive characteristics under 99% compression. Compared with the results of polydisperse surfactants, these results revealed that the structural homogeneity of the surfactant was an important determining factor in the PC gel’s mechanical properties. After washing to remove cytotoxic surfactants, PC gel could be used for biotechnological applications such as scaffold materials in cell cultivation systems. This study established a novel method for creating transparent, durable protein-based gel materials from natural protein sources using monodisperse ionic surfactants. Understanding the molecular mechanism of the origin of the optical transparency of egg white proteins in the thermal gelled state is important for the future application of egg white proteins. Since egg white is a complex system containing various proteins, it is difficult to analyze the physical properties of egg white in solution and in the gelled state. Detailed analysis of the structure-functional properties of egg white proteins in the standard solution and PC states, and their gel states, using purified egg white proteins is necessary for future research. Also, the structural analysis in this study is insufficient to reveal the high-strength nature of PC gels. In the research stage of PC gels using purified proteins, detailed properties of our protein condensate gel could be characterized by various light scattering methods, which are effective for gel structure analysis, and by direct structural observation using electron microscopy and AFM. The properties of protein condensate gel will be better understood through such structural analysis, leading to the developing of novel biomaterials using natural protein resources in the future.

## Methods

### Preparation of pigeon egg white (PEW) solution

PEW was collected from fresh market-purchased pigeon eggs, filtered twice through a mesh, diluted with an equal weight of water, stirred for 30 min at 4 °C to homogenize the solution, and centrifuged (10,000*g* for 10 min). The supernatant was dialyzed twice using a 1000 MW cutoff dialysis bag against water for 12 h to remove small molecular substances and centrifuged (10,000*g* for 10 min) to remove insoluble aggregates. The clear homogenized supernatant was stored at 4 °C. Protein concentration of the PEW solution was measured by BCA method (BCA Protein Assay Kit, Thermo Fisher Scientific, USA) with bovine serum albumin as the standard.

### Synthesis and characterization of surfactants

Polydisperse anionic C_12_E_4.5_ was purchased from Sigma-Aldrich (catalog 463256) and purified, polydisperse cationic C_12_E_4.5_ was synthesized from polydisperse anionic as previously reported^12^. Monodisperse anionic and cationic C_12_E_5_ surfactants were newly synthesized in this study (the detail of the synthesis are described in supplemental information). ES-TOF MS analyses of surfactants were performed with a Q-TOF Micro spectrometer (Waters Corp., USA) in a positive mode.

### Preparation of surfactant solution

Anionic and cationic surfactants were dissolved in MilliQ-grade water. The solutions were neutralized with NaOH and HCl, respectively, and adjusted to 100 mM. The surfactant solutions were stored at room temperature.

### Preparation of PC(PEW-C_12_E_5_) and PC(PEW-C_12_E_4.5_)

Solutions of 100 mM anionic and cationic C_12_E_5_ surfactants were mixed in a volume ratio of 15/85, and 100 μL of the mixed solution was added to 1 mL of 10 mg mL^−1^ PEW solution. The mixed solution was centrifuged (10,000*g* for 2 min), and PC(PEW-C_12_E_5_) was observed at the bottom of the tube as a transparent liquid. The procedure can be scaled up to prepare large quantities. PC(PEW-C_12_E_4.5_) was obtained by adding 80 μL of 100 mM mixture of anionic and cationic C_12_E_4.5_ surfactants (anionic/cationic = 15/85) to 1 mL of 10 mg mL^−1^ PEW solution. The PC formation yield was calculated from the protein concentration (measured by BCA method) of the supernatant after PC formation as follows:

$${\text{Formation yield }}\left( \% \right) \, = { 1}00 \times \left( {{1 } - \, \left[ {{\text{protein}}} \right]_{{{\text{sup}}}} /\left[ {{\text{protein}}} \right]_{0} } \right),$$

where [protein]_0_ is protein concentration of PEW solution before the addition of surfactants (10 mg mL^−1^) and [protein]_sup_ is protein concentration of the supernatant (aqueous layer) after PC formation.

### Preparation of PC-gels

PC(PEW-C_12_E_5_) and PC(PEW-C_12_E_4.5_) were poured into appropriate molds and heated at 50, 60, 70, 80, and 90 °C for 20 min in a water bath or by sandwiching between heat blocks. The prepared gels were stored in water at 4 °C.

### Measurement of protein content of PC

An aliquot of PC(PEW-C_12_E_5_) and PC(PEW-C_12_E_4.5_) was mixed with a ninefold volume of buffer (20 mM Tris–HCl, pH 8.0, containing 200 mM NaCl). Under these electrolyte conditions, the PCs were reconverted to an aqueous protein solution. The protein concentration of the solution was measured by BCA method with bovine serum albumin as the standard. The result was multiplied by ten to determine the protein content of PC.

### Measurement of water content of PC

The PC water content was measured as the difference between the mass weight of the wet and dry samples. The dried samples were prepared in a vacuum at 70 °C for 6 h.

### Measurement of transparency of PC-gels

The transparency of 1-mm thick PC(PEW-C_12_E_5_) and PC(PEW-C_12_E_4.5_) gel was measured at 400–800 nm wavelength using a spectrophotometer (Mapada Corp., Shanghai, China).

### Measurement of mechanical strength of PC-gels

The compressive stress–strain curve was measured using an INSTRON 5940 (Instron Corp., Norwood, MA, USA). Cylindrical gel samples 8.5 mm diameter and 8.0 mm thick were compressed at a 1.2 mm min^−1^ strain rate. The tensile stress–strain measurements were performed using an INSTRON 5943. The dumbbell-shaped specimens with a length of 50 mm, narrowest breadth of 5 mm, and tickness of 1 mm were stretched at a 4 mm min^−1^ strain rate.

### Evaluation of the effect of PC(C_12_E_5_)-gel washing on cell toxicity

PC(PEW-C_12_E_5_)-gel was incubated at room temperature in a mixture of ethanol and phosphate buffer saline (PBS) at 20/80, 30/70, 40/60, and 50/50 (v/v) ratios. The ethanol/PBS mixtures were exchanged with fresh solutions five times every 12 h during the gel-washing process in the 72-h incubation period. The incubated gels were immersed in 75% ethanol for 1 h and PBS for 2 h and then used as washed gels in the following cytotoxicity test.

### PC gel cytotoxicity evaluation using HL-60 cells

Human promyelocytic leukemia cells (HL-60) (iCell Bioscience Inc., Shanghai, China) in Iscove’s Modified Dulbecco’s Medium (IMDM) and 20% fetal bovine serum (FBS) medium were cultured in a CO_2_ incubator at 37 °C with 5% CO_2_. Two milliliters of 5 × 10^5^ cells mL^−1^ culture medium cell density were added to 3.5 cm diameter Petri dishes and cultured at 37 °C with 5% CO_2_ for 48 h in the presence of a 20 mm × 15 mm × 1 mm piece of washed gel. One hundred microliters of the culture were collected every 12 h, mixed with 10 μL of CCK-8 reagent (Cell Counting Kit-8, Dojindo, Inc., Kumamoto, Japan), incubated for 3 h in a CO_2_ incubator, and the absorbance at 450 nm was measured. All experiments were performed in triplicate and averaged. The same experiment was performed in the absence of a piece of the gel as a positive control group.

### Cell culture on gel surface

Cell growth on the surface of the planer-shaped PC(PEW-C_12_E_5_)-gel was observed. The planar-shaped PC(PEW-C_12_E_5_)-gel (20 × 15 × 1 mm) was washed as described above using a 40/60 ethanol/PBS ratio. The washed gel was placed in a 3.5 cm Petri dish. Three milliliters of NCI-H460 cells suspended in RPMI 1640 and 10% FBS medium 2 × 10^5^ cells mL^−1^ density was seeded in the dish and cultured in a CO_2_ incubator at 37 °C with 5% CO_2_ for 96 h. The culture medium was replaced with fresh medium at the time of 48 h. The same experiment was performed without the gels, and cells were cultured on a tissue culture-treated culture dish (Corning Inc., #430165). Cells were observed at 48 and 96 h using a microscope (Eclipse Ti2-U, Nikon, Japan, CCD camera DS-Ri2, Nikon, Japan). 96 h-cultivation cells were stained with -Cellstain- Double Staining Kit (Dojindo, Inc., Kumamoto, Japan), and the living cells were observed using a fluorescent microscope (Eclipse Ti2-U, Nikon, Japan, CCD camera DS-Ri2, Nikon, Japan).

## Supplementary Information

Supplementary Information.

## Data Availability

All data generated or analyzed during this study are included in this published article (and its supplementary information files).
