# Supplementary material for: Pigeon egg white protein-based transparent durable hydrogel via monodisperse ionic surfactant-mediated protein condensation
Source: Sci Rep. 2022 Mar 17;12:4633. doi: 10.1038/s41598-022-08375-x (PMC8930986; doi:10.1038/s41598-022-08375-x)
Supplement: Supplementary file 1 — Supplementary Information. [file 41598_2022_8375_MOESM1_ESM.pdf]

Supplementary information for

Pigeon egg white protein-based transparent durable hydrogel *via* monodisperse ionic surfactants-mediated protein condensation.

Xinlian Zhou, Zaozao Chen and Tatsuya Nojima

Contents:

1. Synthesis of anionic and cationic $C_{12}E_5$ surfactants
2. Transparency of the PC(PEW-$C_{12}E_{4.5}$)-gel
3. Cytotoxicity test of the washed gel on NCI-H460 cell line
4. Compression test of the PC(PEW-$C_{12}E_5$)-gel after washing out of surfactants
5. The full-length SDS-PAGE gel used in Figure 1c (Figure S4)

1. Synthesis of anionic and cationic $C_{12}E_5$ surfactants

^{1}H NMR spectra were recorded using a AVANCE 500 FT-NMR spectrometer (Bruker, USA) at 500 MHz. ^{1}H chemical shifts were referenced to tetramethylsilane.

Hexa(ethylene glycol) trityl ether (**1**)

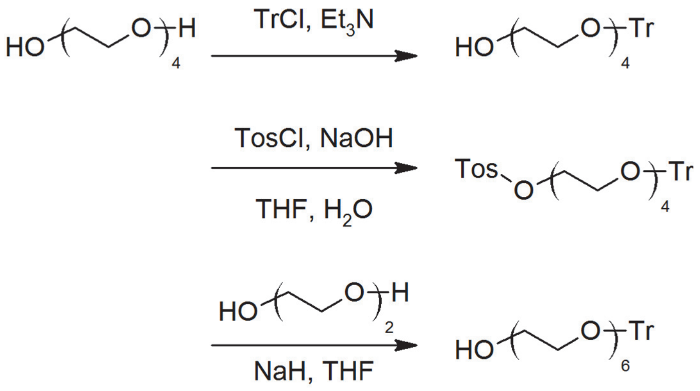

Hexa(ethylene glycol) trityl ether (**1**) was synthesized in 3 steps according to the reported method for synthesis of octa(ethylene glycol) trityl ether (Reference no. 13 and 14 in main manuscript).

Trityl chloride (TrCl) (25.0 g, 89.7 mmol, 1eq) was added over 15 minutes to the solution of tetra(ethylene glycol) (112g, 577 mmol) and triethylamine (11.6 g, 115 mmol) in 100 mL toluene at room temperature under argon atmosphere. The mixture was stirred at room temperature for 3 hours, diluted with ethyl acetate (100 mL), and vacuum-filtered. The filtrate was washed with water (2 × 150 mL, 3 × 50 mL), saturated ammonium chloride aq. (2 × 50 mL) and brine (50 mL), dried over sodium sulfate and evaporated to give crude tetra(ethylene glycol) trityl ether.

Crude tetra(ethylene glycol) trityl ether was dissolved in THF (120 mL) and cooled for 30 min on a water-ice bath. To the solution was added a sodium hydroxide solution (12.5 g, 313 mmol in 40 mL water). To the resulting heterogeneous mixture, the solution of *p*-toluenesulfonyl chloride (TosCl) (17.0 g, 80.2 mmol in 40 mL THF) was added dropwise over 15 min. The mixture was stirred for 5 hours on a water-ice bath and for 16 hours at 25 °C. The reaction mixture was diluted with water (30 mL) and methyl t-butyl ether (100 mL). The organic layer was washed with water (50 mL) and brine (2 × 50 mL), dried over sodium sulfate and evaporated to give crude tetra(ethylene glycol) trityl ether *p*-toluenesulfonate.

Crude tetra(ethylene glycol) trityl ether *p*-toluenesulfonate was dissolved in dry THF (40 mL) and added dropwise over 15 min to a suspension of sodium hydride

(hexane-washed, 120 mmol in 100 mL THF) cooled on water-ice bath under argon atmosphere. To the resulting mixture was added di(ethylene glycol) (63.7 g, 600 mmol) over 5 min and reflux for 4 hours. The reaction mixture was washed with water-brine mixture (1:1 v/v, 300 mL and 2 × 100 mL) and brine (50 mL), dried over sodium sulfate, and evaporated. The residue was purified by column chromatography on silica gel (eluted with 1:9 hexane/EtOAc, EtOAc, then 9:1 EtOAc/MeOH) to give the title compound (31.8 g, 60.7 mmol, 68 % in 3 steps). ^{1}H NMR (500 MHz, $CDCl_3$, δ): 7.46 (d, 6H), 7.31–7.20 (m, 9H), 3.72–3.62 (m, 20H), 3.59 (t, 2H), 3.23 (t, 2H), 2.51 (b, 1H, OH).

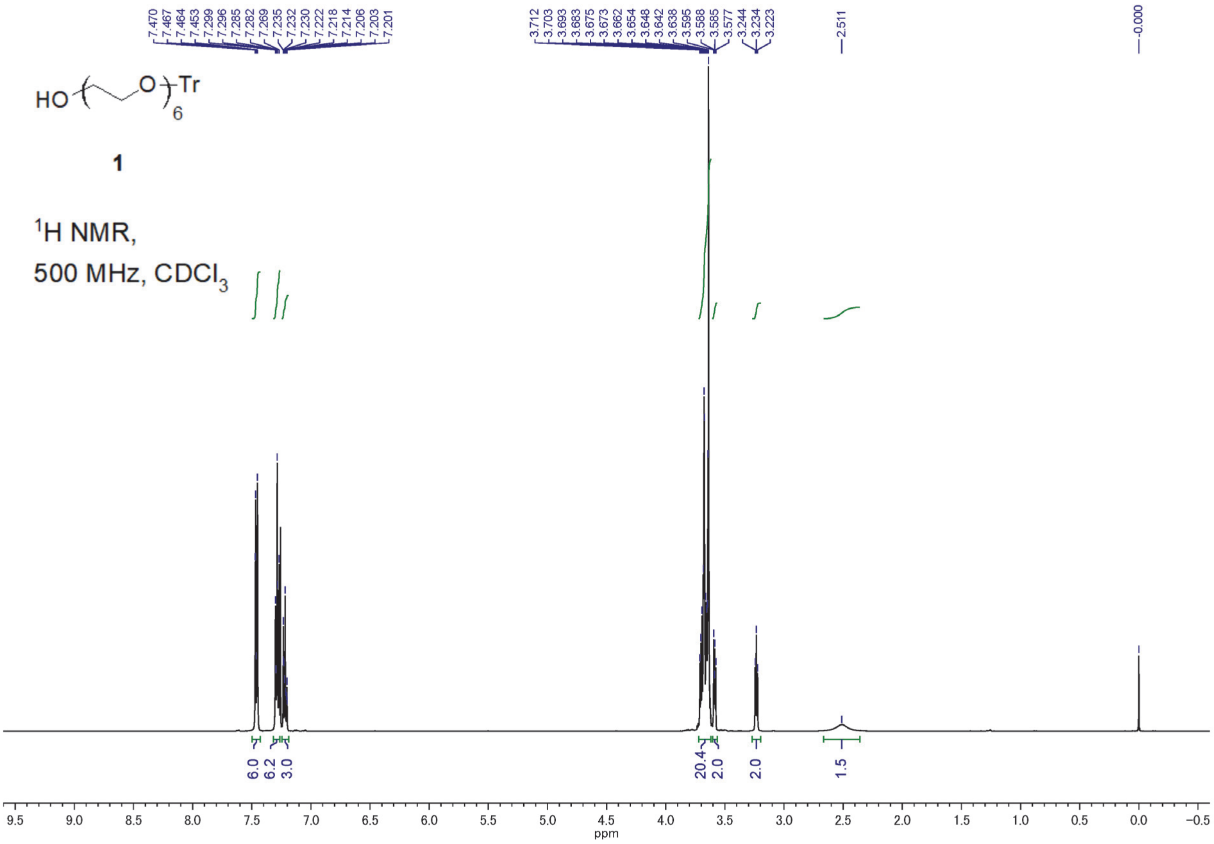

<u>Hexa(ethylene glycol) dodecyl ether (**2**)</u>

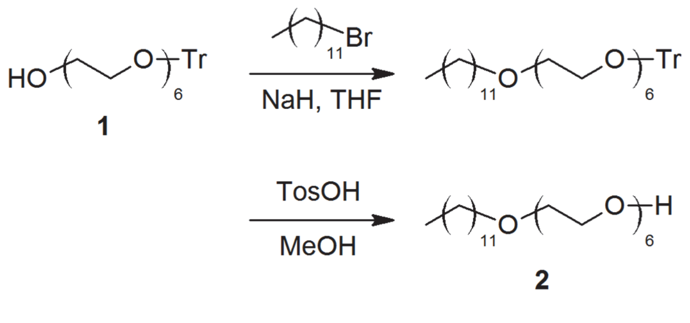

Hexa(ethylene glycol) trityl ether (**1**) (26.2 g, 50 mmol) was dissolved in dry THF (50 mL) and added dropwise over 15 min to a suspension of sodium hydride (hexane-washed, 100 mmol in 100 mL THF) cooled on water-ice bath under argon atmosphere. To the resulting mixture was added 1-Bromo dodecane (15.0 g, 60 mmol) and reflux for 4 hours. The reaction mixture was diluted with EtOAc (100 mL) and washed with water-brine mixture (1:1 v/v, 100 mL and 2 × 50 mL) and brine (50 mL), dried over sodium sulfate, and evaporated to give crude Hexa(ethylene glycol) dodecyl trityl ether.

Crude Hexa(ethylene glycol) dodecyl trityl ether was suspended in methanol (100 mL). *p*-Toluenesulfonic acid monohydrate (TosOH) (0.38 g, 2 mmol) was added, and the mixture was stirred for 18 hours. Crushed ice (about 12 g) was slowly added to the reaction mixture until a white crystalline was precipitated in the mixture. Water (150 mL) was added over 5 min, and the mixture was cooled on a water-ice bath, then vacuum-filtered. The filtrate was concentrated with a rotary evaporator to remove methanol and extracted with CH_2Cl_2 (100 mL, 50 mL and 30 mL). The extracted CH_2Cl_2 solution was washed with brine (50 mL), dried over sodium sulfate, and evaporated. The residue was purified by column chromatography on silica gel (eluted with EtOAc, then 9:1 EtOAc/MeOH) to give the title compound (17.1 g, 37.9 mmol, 76 % in 2 steps). ^{1}H NMR (500 MHz, $CDCl_3$, δ): 3.74–3.71 (m, 2H), 3.70–3.56 (m, 22H), 3.44 (t, 2H), 2.72 (t, 1H, OH), 1.65–1.50 (m, 2H), 1.37–1.19 (b, 18H), 0.88 (t, 3H).

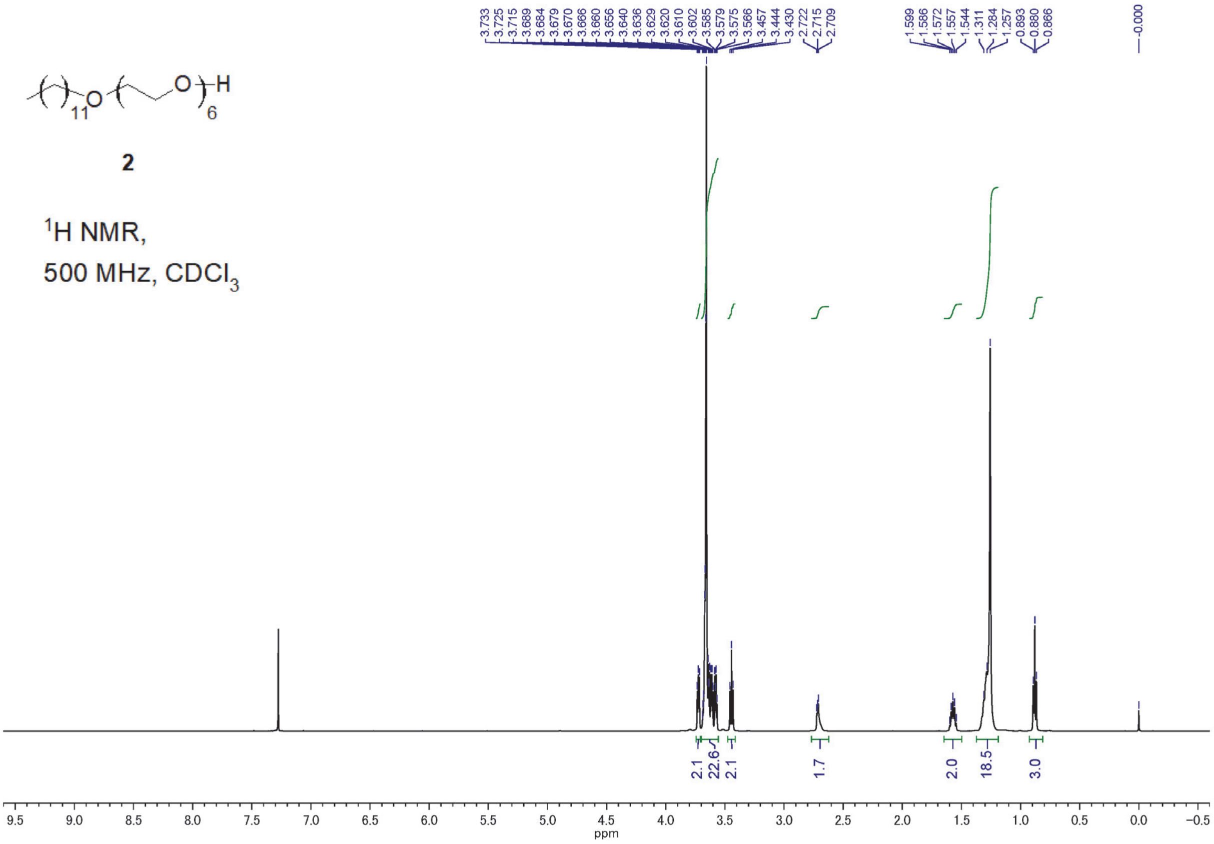

<u>Glycolic acid penta(ethylene glycol) dodecyl ether (**3**)</u>

Compound **3** is referred to as an “anionic $C_{12}E_5$” in the manuscript.

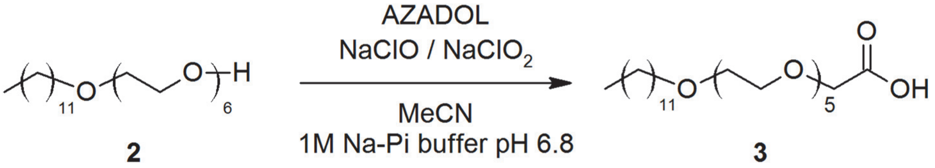

Hexa(ethylene glycol) dodecyl ether (**2**) (2.7 g, 6.0 mmol) was dissolved in acetonitrile (15 mL) and 1.0 M sodium phosphate buffer pH 6.8 (15 mL). To the mixture was added the oxidation catalyst 2-Hydroxy-2-azaadamantane (AZADOL) (18.4 mg, 0.12 mmol) followed by $NaOCl\cdot 5H_2O$ (19.7 mg, 0.12 mmol) and then $NaClO_2$ (80%) (1.09 g, 12 mmol). After completion of the reaction (~20 hours), 1 mL ethanol was added and stirred for 2 hours to quench the reaction, and 0.8 mL of 37% HCl was added to the mixture. The aqueous layer was discarded, and the organic layer was washed with brine (2 × 20 mL) and evaporated. The residue was co-evaporated with toluene and 1.4-dioxane, dissolved in CH_2Cl_2, dried over sodium sulfate, and evaporated. The residue was purified by column chromatography on aminopropyl modified silica gel (eluted with acetone, then acetone containing 5% HCl (prepared by

the mixing of acetone and 37 % HCl aq.)) to give the title compound (2.3 g, 5.0 mmol, 83 %). ^{1}H NMR (500 MHz, $CDCl_3$, δ): 4.16 (s, 2H), 3.78–3.73 (m, 2H), 3.73–3.62 (m, 16H), 3.61–3.56 (m, 2H), 3.45 (t, 2H), 1.60–1.54 (m, 2H), 1.38–1.19 (b, 18H), 0.88 (t, 3H).

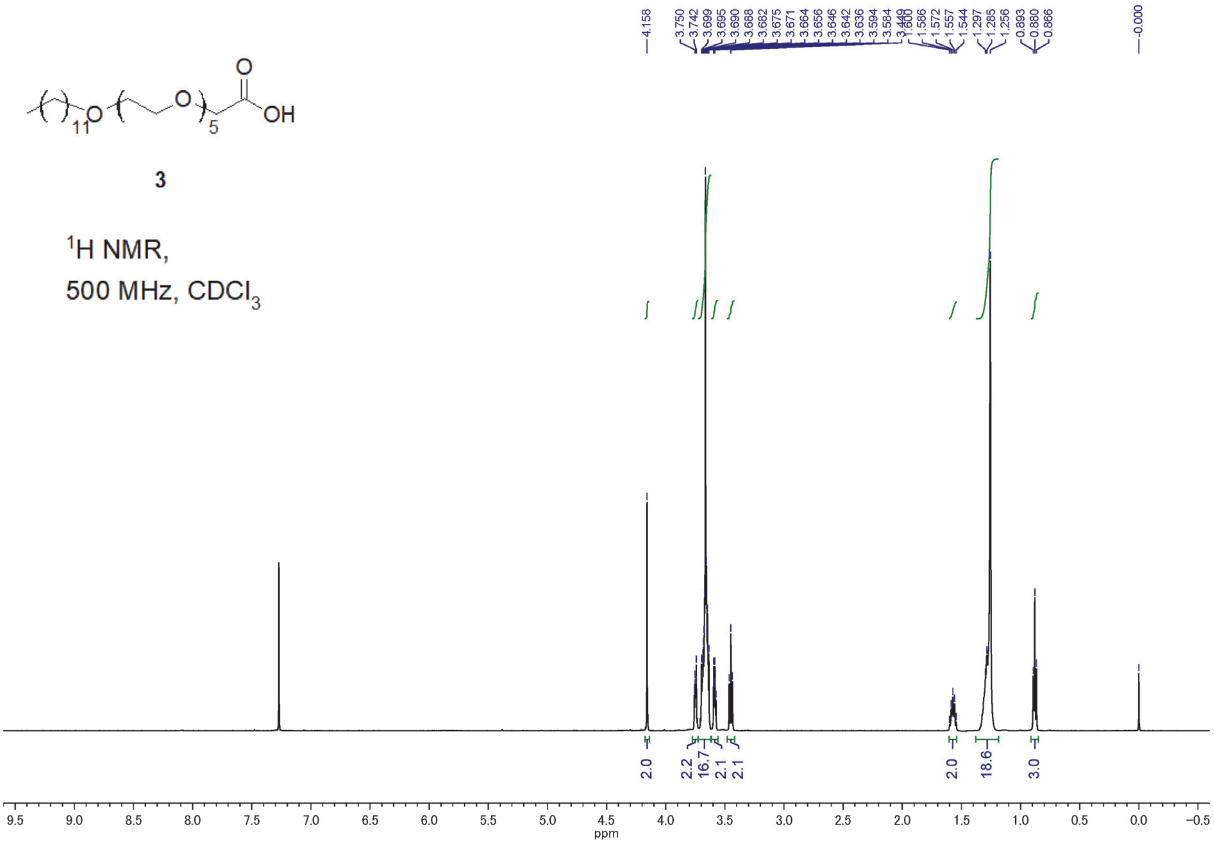

2-Azidoethoxy penta(ethylene glycol) dodecyl ether (**4**)

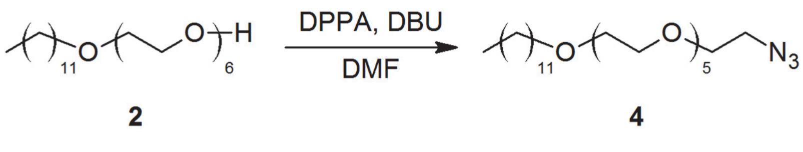

Hexa(ethylene glycol) dodecyl ether (**2**) (9.0 g, 20.0 mmol) and diphenylphosphoryl azide (DPPA) (16.5 g, 60 mmol) was dissolved in dry DMF (40 mL). To the mixture was added 1,8-diazabicyclo[5.4.0]undec-7-ene (DBU) (9.1 g, 60 mmol) and stirred at 90 °C for 3 hours. The reaction mixture was diluted with water (120 mL), extracted with hexane-EtOAc (1/1 v/v, 100 mL), washed with water (2 × 80 mL), saturated ammonium chloride aq. (2 × 40 mL) and brine (40 mL), dried over sodium sulfate, and evaporated. The residue was purified by column chromatography on

silica gel (eluted with 1:1 hexane-EtOAc, then a gradient to 100 % EtOAc) to give the title compound (6.0 g, 12.6 mmol, 63 %). ^{1}H NMR (500 MHz, $CDCl_3$, δ): 3.70–3.61 (m, 20H), 3.61–3.55 (m, 2H), 3.44 (t, 2H), 3.39 (t, 2H), 1.62–1.54 (m, 2H), 1.37–1.19 (b, 18H), 0.88 (t, 3H).

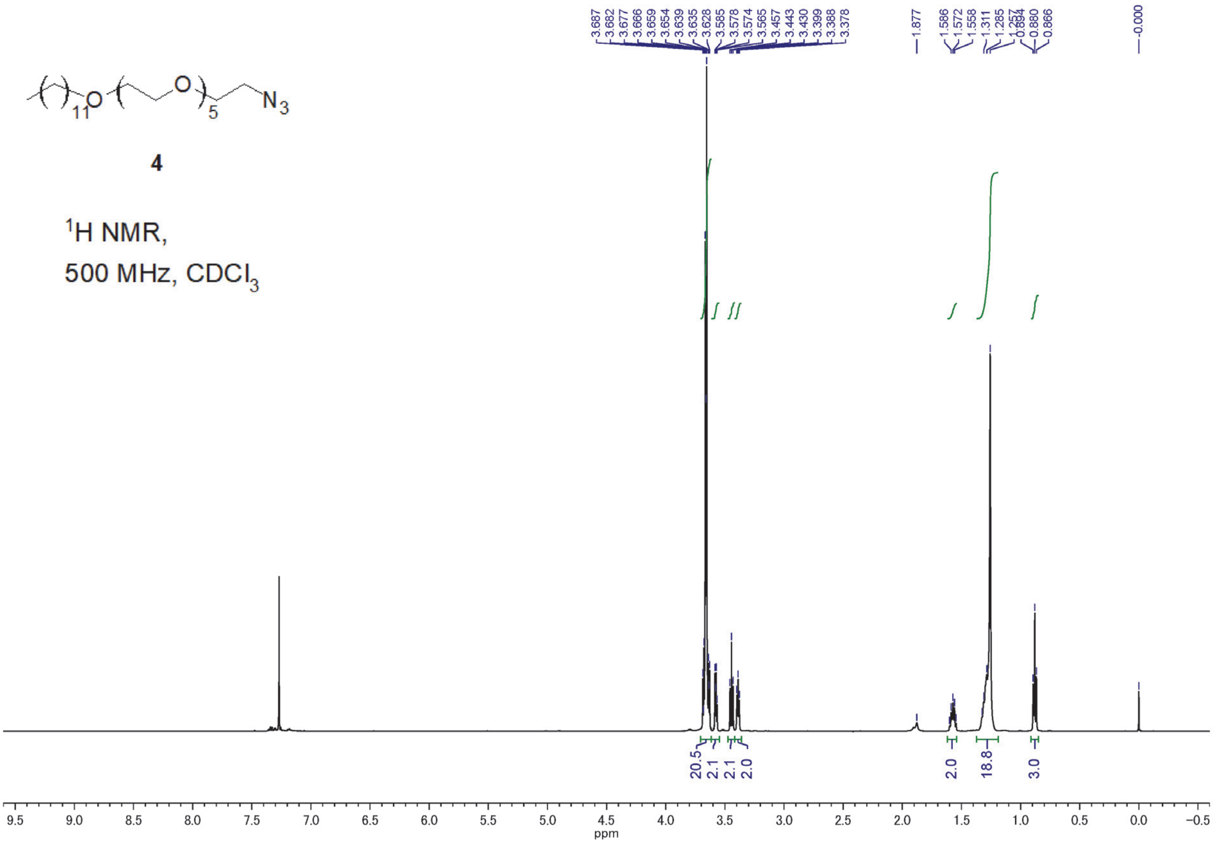

2-Aminoethoxy penta(ethylene glycol) dodecyl ether (**5**)

Compound **5** is referred to as an "cationic $C_{12}E_5$" in the manuscript.

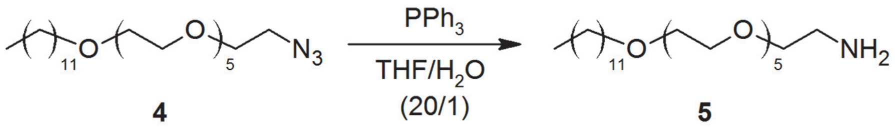

2-Azidoethoxy penta(ethylene glycol) dodecyl ether (**4**) (5.7 g, 12 mmol) was dissolved in THF (30 mL) and water (1.5 mL). To the solution was added triphenylphosphine (PPh_3) (6.3 g, 24 mmol) and stirred at room temperature for 24 hours. The reaction mixture was concentrated and purified by column chromatography on silica gel (eluted with 9:1 EtOAc-MeOH, 9:1 CH_2Cl_2-MeOH, then 9:1 CH_2Cl_2-MeOH with 1 % triethylamine) to give the title compound (4.8 g, 10.7 mmol, 89 %). ^{1}H

NMR (500 MHz, $CDCl_3$, δ): 3.68–3.61 (m, 18H), 3.60–3.56 (m, 2H), 3.53 (t, 2H), 3.44 (t, 2H), 2.88 (t, 2H), 2.10 (s, 2H), 1.62–1.51 (m, 2H), 1.39–1.17 (b, 18H), 0.88 (t, 3H).

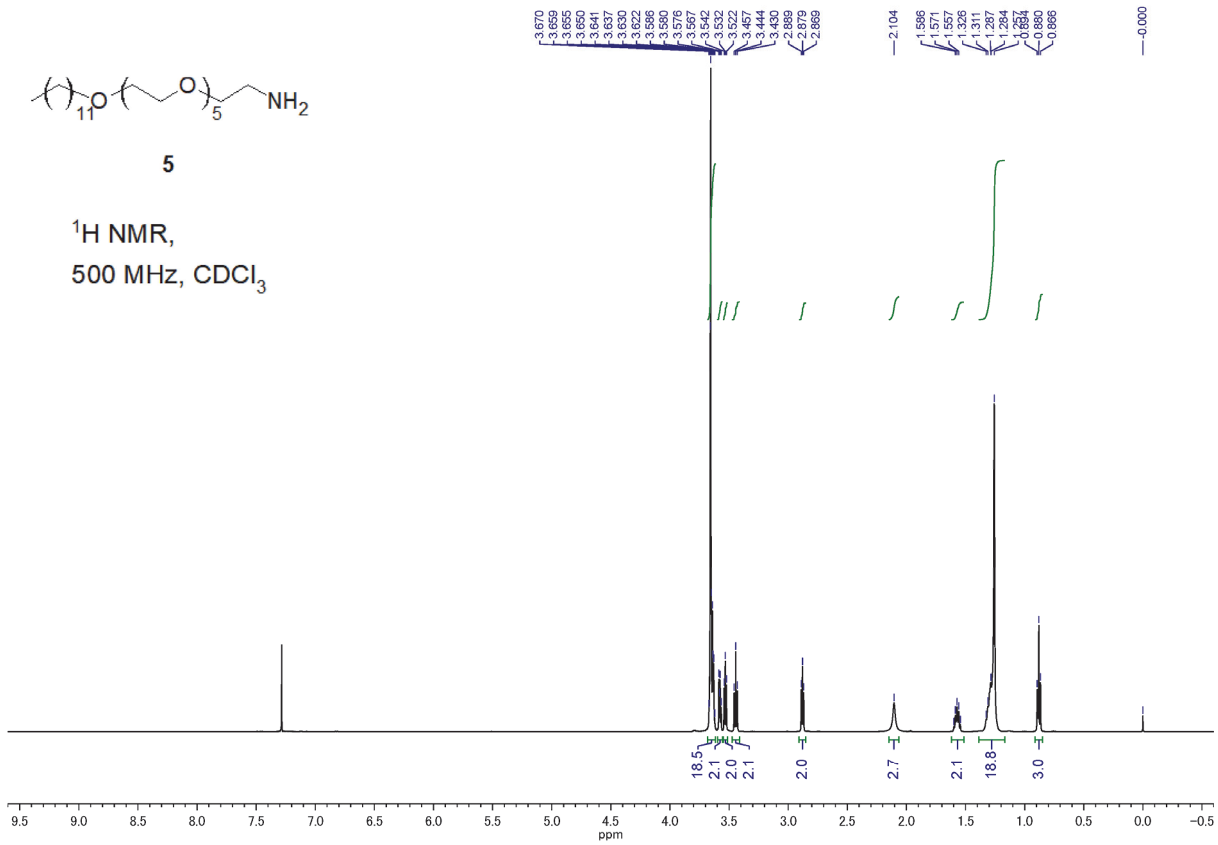

2. Transparency of the PC(PEW-$C_{12}E_{4.5}$)-gel

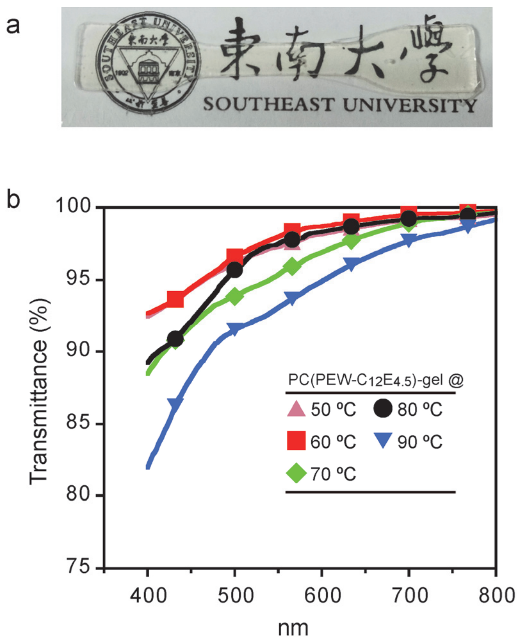

Fig S1. (a) PC(PEW-$C_{12}E_{4.5}$)-gel prepared at 70 °C (b) Transparency measurement of the of PC(PEW-$C_{12}E_{4.5}$)-gel with the thickness of 1 mm.

3. Cytotoxicity test of the washed gel on NCI-H460 cell line

In this experiment, the optimal washing condition (ethanol/PBS = 40/60) determined from the result of the cytotoxicity test on HL-60 was used. 100 μL of NCI-H460 cells (Exposure of human non-small cell lung cancer cell, Cobioer Bioscience CO., Ltd, Nanjing, China) suspended in the medium (RPMI + 10% FBS) at the cell density of 3×10^4 cells mL^{-1} was added to 96-well plate. After 24 hours of cultivation in a CO_2 incubator at 37 °C with 5% CO_2, a peace of 25 mg of the washed gel was added and cultured at 37 °C with 5% CO_2. Culture medium was exchanged to fresh medium at 48 hours. The gel was removed from the well at 6, 12, 24, 36, 46 and 96 hours, and the culture medium was exchanged to fresh medium. 10 μL of CCK-8 reagent was added to the well and incubated at 37 °C with 5% CO_2. The same experiment without an adding of the gel was performed as a positive control group. The absorbance at 450 nm was measured using a microplate reader and plotted using empty well as a baseline. All experiments were performed three times and averaged.

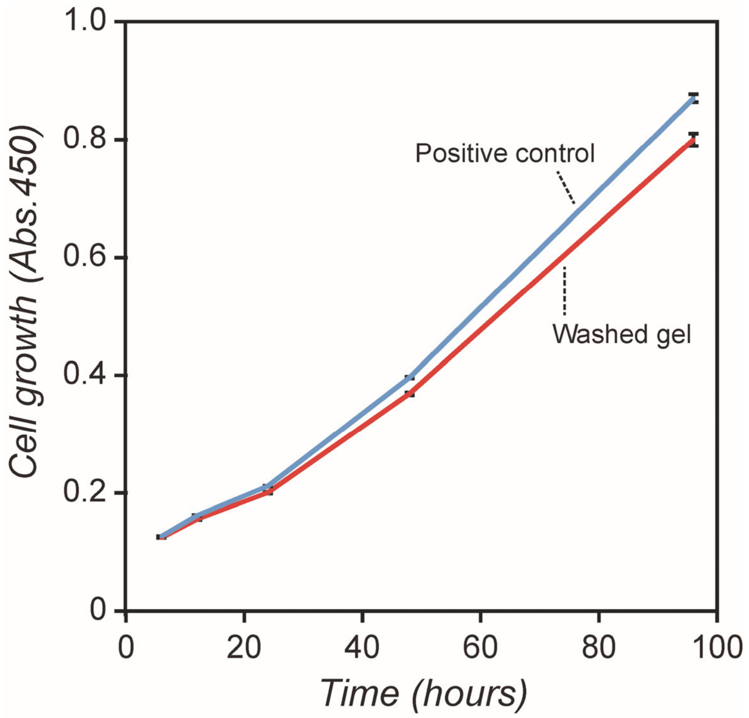

Fig S2. Growth of NCI-460 cells with the washed PC(PEW-$C_{12}E_5$)-gels. Growth of NCI-H460 cells in the presence of PC(PEW-$C_{12}E_5$)-gel washed in the mixture of ethanol/PBS at the ratio of 40/60 was comparable to that of the positive control group, indicating the non-cytotoxicity of the gel. Error bars indicate S.D.

4. Compression test of the washed PC(PEW-$C_{12}E_5$)-gel

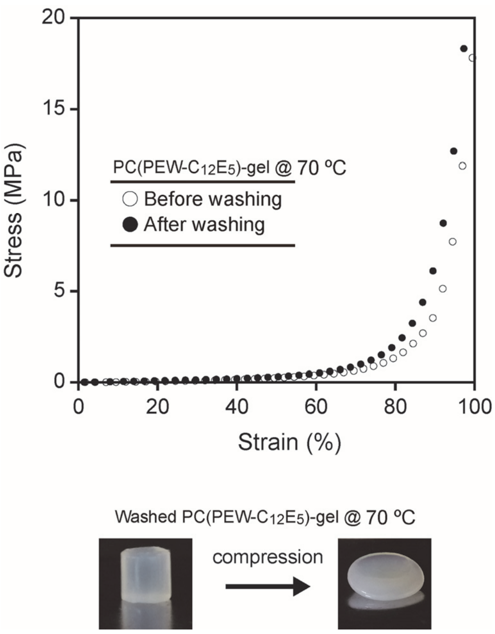

Fig S3. Compression stress-strain curve of washed PC(PEW-$C_{12}E_5$)-gel. PC(PEW-$C_{12}E_5$)-gel washed in the mixture of ethanol/PBS at the ratio of 40/60 showed an almost equal compressive strength to that of before washing, and the unbroken property on a compression.

5. **The full-length SDS-PAGE gel used in Figure 1c**

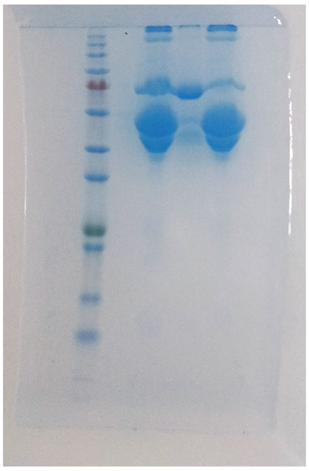

Fig S4. The full-length SDS-PAGE gel image used in Figure 3c before gray scaling. RealBand 3-color High Range Protein Marker (# C620014, Sangon Biotech, Shanghai) was used as molecular weight marker (leftmost lane).The Gel was stained with CBB.
